# Modification of β-Defensin-2 by Dicarbonyls Methylglyoxal and Glyoxal Inhibits Antibacterial and Chemotactic Function *In Vitro*

**DOI:** 10.1371/journal.pone.0130533

**Published:** 2015-08-05

**Authors:** Janna G. Kiselar, Xiaowei Wang, George R. Dubyak, Caroline El Sanadi, Santosh K. Ghosh, Kathleen Lundberg, Wesley M. Williams

**Affiliations:** 1 Center for Proteomics and Bioinformatics, Case Western Reserve University, Cleveland, Ohio, United States of America; 2 Department of Periodontics, Case Western Reserve University, Cleveland, Ohio, United States of America; 3 Department of Physiology and Biophysics, Case Western Reserve University, Cleveland, Ohio, United States of America; 4 Department of Biological Sciences, Case Western Reserve University, Cleveland, Ohio, United States of America; University of Kiel, GERMANY

## Abstract

**Background:**

Beta-defensins (hBDs) provide antimicrobial and chemotactic defense against bacterial, viral and fungal infections. Human β-defensin-2 (hBD-2) acts against gram-negative bacteria and chemoattracts immature dendritic cells, thus regulating innate and adaptive immunity. Immunosuppression due to hyperglycemia underlies chronic infection in Type 2 diabetes. Hyperglycemia also elevates production of dicarbonyls methylgloxal (MGO) and glyoxal (GO).

**Methods:**

The effect of dicarbonyl on defensin peptide structure was tested by exposing recombinant hBD-2 (rhBD-2) to MGO or GO with subsequent analysis by MALDI-TOF MS and LC/MS/MS. Antimicrobial function of untreated rhBD-2 vs. rhBD-2 exposed to dicarbonyl against strains of both gram-negative and gram-positive bacteria in culture was determined by radial diffusion assay. The effect of dicarbonyl on rhBD-2 chemotactic function was determined by chemotaxis assay in CEM-SS cells.

**Results:**

MGO or GO *in vitro* irreversibly adducts to the rhBD-2 peptide, and significantly reduces antimicrobial and chemotactic functions. Adducts derive from two arginine residues, Arg^22^ and Arg^23^ near the C-terminus, and the N-terminal glycine (Gly^1^). We show by radial diffusion testing on gram-negative *E*. *coli* and *P*. *aeruginosa*, and gram-positive *S*. *aureus*, and a chemotaxis assay for CEM-SS cells, that antimicrobial activity and chemotactic function of rhBD-2 are significantly reduced by MGO.

**Conclusions:**

Dicarbonyl modification of cationic antimicrobial peptides represents a potential link between hyperglycemia and the clinical manifestation of increased susceptibility to infection, protracted wound healing, and chronic inflammation in undiagnosed and uncontrolled Type 2 diabetes.

## Introduction

Human β-defensin peptides (hBDs) are an evolutionarily conserved group of cationic, low molecular weight, unglycosylated peptides crucial to the antimicrobial and cell signaling functions of the innate immune system [1, 2]. In mammals, including humans, the principle classes of the peptide are the α- and β-defensins. Members of both classes exhibit structural similarities, the presence of six cysteine residues forming three intramolecular disulfide bonds, and an unusually high number of arginine and lysine residues [3, 4]. Whereas α-defensins are expressed by blood-borne cells such as neutrophils [5], the hBDs are secreted predominantly by integumentary, lung, urogenital, oral and intestinal epithelium [4–6]. The hBD-2 peptide is inducible, and is expressed by epithelium and epithelial-derived cells [7], although recently expression of the peptide by vascular endothelium associated with oral squamous cell carcinoma has been reported [8]. In addition to their antimicrobial function hBDs also are important to regulation of innate and adaptive immune response and inflammation [9–11]. HBD-2 responds primarily to gram-negative bacteria, and modulates inflammation in humans [12] by inducing cellular expression of both pro- and anti-inflammatory cytokines and chemokines, including IL-6, IL-10, MIP-3α, MCP-1, and RANTES through activation of G protein-coupled CCR6 and PLC-dependent pathways [13]. Moreover, hBD-2 is instrumental in promoting immune cell migration and proliferation, angiogenesis, chemotaxis, and wound repair through phosphorylation of the epithelial cell growth factor receptor (EGFR), and signal transducer and activator of transcription (STAT) 1, and STAT3 [13,14].

Chronic hyperglycemia is most often associated with the onset of Type 2 diabetes mellitus, but reduced tissue utilization of glucose and resultant elevation of blood glucose can also occur with normal aging [15,16], chronic disease, such as Alzheimer’s disease [17,18], and with chronic wounds [19]. A consequence of chronically elevated glucose is the increased production, and reduced degradation of dicarbonyl molecular species, including methylglyoxal (MGO) and glyoxal (GO) [20]. Although both are highly reactive α-oxoaldehydes that target arginine (Arg), lysine (Lys), and cysteine (Cys) residues of susceptible proteins, MGO is generally the more reactive [21,22]. These reactions however are selective, thus the presence of Arg, Lys or Cys residues within a protein does not necessarily lead to adduction [23]. All studied hBD peptides contain multiple Arg, Lys, and Cys residues. In hBDs the Cys residues form three intramolecular disulfide bonds considered fundamental to maintenance of hBD tertiary structure, and resistance to attack by proteases [24], while Arg and Lys residues are believed to contribute to disruption of the bacterial cell wall [25]. Dicarbonyl-induced modification of these residues through the formation of irreversible Advanced Glycation End products (AGEs) has the potential to significantly impair both antimicrobial and/or immunomodulatory functions of not only the hBDs, but other cationic antimicrobial peptides containing multiple arginine or lysine residues.

In an earlier study on the effect of scratch-wounding and high glucose on *Fusobacterium nucleatum*-induced epithelial cell expression of hBD mRNA we observed by RT-qPCR that the addition of 30 mM glucose to cultured cells significantly reduced expression of hBD-2 mRNA (unpublished, S1 Fig). Our findings were subsequently confirmed by Lan et al. [26]. These initial observations led us to hypothesize that since dicarbonyl molecular species, such as MGO have been shown to form crosslinks between the guanine residue of template DNA and susceptible amino acid residues of DNA polymerase [27], and that MGO may also cause mRNA instability [28], perhaps dicarbonyls could also affect hBD function by direct modification of the peptide. We reasoned that since dicarbonyls exhibit high reactivity with Arg and Lys residues [29], and cationic antimicrobial peptides, including hBDs contain unusually high numbers of both residues there would be a higher likelihood that irreversible modification of these peptides would occur. In the present study recombinant hBD-2 (rhBD-2) was exposed to approximated physiological concentrations of MGO and GO and the peptide tested for antimicrobial activity and chemotactic function. We show that exposure of rhBD-2 to dicarbonyls significantly attenuates both antimicrobial and chemotactic function *in vitro*. Our findings suggest that under hyperglycemic conditions *in vivo* functionality of cationic antimicrobial peptides, in general, may be impaired as a result of carbonyl adduction and irreversible modification of susceptible amino acid residues. Our findings describe a previously unreported mechanism by which chronic hyperglycemia may increase susceptibility to chronic infection, and delayed wound repair in cases of uncontrolled or poorly controlled hyperglycemia.

## Materials and Methods

### Materials

Recombinant hBD-2 (rhBD-2) (cat. no. 300–49) was purchased from PeproTech (Rocky Hill, NJ). Purity of the recombinant was 98% as determined by SDS-PAGE gel and HPLC analysis. Activity of the peptide was determined by its ability to attract immature dendritic cells when tested by the manufacturer within a concentration range of 10 to 100 ng/ml. Glyoxal (cat. no. 50660) was purchased from Sigma-Aldrich (St. Louis, MO). Methylglyoxal was a kind gift courtesy of Dr. Ram Nagaraj (Case Western Reserve University, Cleveland, OH). CEM-SS cells were obtained through the AIDS Research and Reference Reagent Program, Division of AIDS, NIAID, NIH: CEM-SS (Cat # 776) from Dr. Peter L. Nara [30, 31, 32]. This human (Caucasian) acute T4-lymphoblastoid leukemia cell line was initially derived by G.E. Foley et al. and biologically cloned by P.l. Nara et al, as cited.

### Methods

#### Preparation of rhBD-2-dicarbonyl adducts for MALDI TOF MS and LC/MSMS Analysis

rhBD-2 (200 ng/10 μl) was incubated at 37°C with GO or MGO diluted in phosphate-buffered saline (pH 7.5) to a final concentration of 1, 10, or 100 μM. Recombinant hBD-2 (200 ng/10 μl) incubated with phosphate-buffered saline only was used for comparison of mass spectra obtained from samples exposed to the dicarbonyl molecular species. Incubation times were set at 2, 24, 48 and 72 hr. After incubation the mass spectra for each sample was determined by MALDI-TOF MS, and compared for presence of dicarbonyl-induced adducts.

#### MALDI-TOF mass spectroscopy

Samples containing rhBD-2 protein was incubated with MGO or GO for 2, 24, 48 or 72 h, and analyzed by matrix-assisted laser desorption/ionization-time of flight (MALDI-TOF) mass spectrometry (MS). To quench MGO and GO chemical reactions, each sample was purified by solid-phase extraction using C18 pack disposable pipette tips (ZipTip C18 Millipore, Co.). Extracted samples were then mixed with α-Cyano-4-hydroxycinnamic acid matrix solution (5mg/ml in 50% acetonitrile containing 0.1% TFA) at the protein to matrix ratio of 1:5 (v/v). MALDI-TOF MS analysis was performed on a prOTOF2000 time-of-flight mass spectrometer (PerkinElmer Co., Boston, MA) equipped with a 337 nm nitrogen laser operating in the positive ion mode with an accelerating voltage of -16 kV, de-clustering potential of 30V, 20 Hz laser rates, and cooling and focusing gas flow rates of 190 and 212 ml/min, respectively. Spectra were acquired by averaging the scans of 500 laser shots to improve data quality and ion statistics. Mass spectra were calibrated externally using the singly protonated ions of angiotensin II and human adrenocorticotropic hormone fragment 7–38.

Reduced and alkylated MGO- and GO-rhBD-2 incubates were analyzed by liquid chromatography-tandem mass spectrometry (LC-MS/MS) to identify the specific site(s) of MGO and GO modification.

#### Proteolysis and MS Analysis

Samples containing rhBD-2 protein and incubated with MGO or GO, were purified by solid-phase extraction using packed disposable ZipTip C18 pipette tips (Millipore, Billerica, MA) according to the manufacturer’s protocol. Extracted samples were then dried and re-suspended in 25 mM ammonium bicarbonate buffer (pH 7.8). The protein samples then were reduced with 5 mM dithiothreitol at 56°C for 45 min, followed by alkylation with 10 mM iodoacetamide in the dark for 45 min to reduce disulfide bridges. Subsequently reduced and alkylated samples were subjected to proteolysis overnight at 37°C by modified trypsin (Promega, Madison, WI) at an enzyme-to-protein ratio of 1:20 wt/wt. The digest mixtures (approximately 400 ng) were loaded onto a 300 μm × 5 mm C18, PepMap reverse phase trapping column to preconcentrate and wash away excess salts using a nano HPLC UltiMate-3000 (Dionex, Sunnyvale, CA) column switching technique. The reverse phase separation was performed on a 75 μm × 15 cm (3um, 100A) Acclaim C18 column (Dionex) using a linear gradient of 5–50% B over 60 min [Buffer A: 100% water/0.1% formic acid (FA); Buffer B: 80% water CAN/0.1% FA]. Proteolytic peptides eluting from the column were directed to an LTQ-FT mass spectrometer (Thermo Fisher Scientific, Fremont, CA) equipped with a nanospray ion source and with the needle voltage of 2.4 kV. All mass spectra were obtained from data-dependent experiments. MS and tandem MS spectra were acquired in the positive ion mode with a full scan MS recorded in the FT analyzer at resolution *R* of 100,000 followed by MS/MS of the eight most-intense peptide ions in the LTQ analyzer. The resulting MS2 data were searched against hBD-2 protein database using Mass Matrix software to identify all specific sites of modification [33]. In particular, MS2 spectra were searched for tryptic peptides of hBD-2 using mass accuracy values of 15 ppm and 0.8 Daltons for MS1 and MS2 scans respectively, with the allowed variable modifications including carbamidomethylation for cysteines, MGO and dehydrated MGO modifications for arginines, lysines and N-terminal amino acid, and three missed cleavage sites. In addition, all detected MS2 spectra for each site of modification were manually verified. The fraction of modified Arg^22^ and Arg^23^ (peptides 11–23 and 23–36, respectively) were calculated from the ratio of the area under ion signals for the modified peptides to the sum of the areas for the unmodified peptides and their modified products.

#### Radial diffusion assay and determination of CFU and rhBD-2 bactericidal activity

Since MGO was found to be more reactive than GO in adducting to hBD only the effect of MGO on rhBD-2 antimicrobial function was tested. The agar-based radial diffusion assay described by Steinberg and Lehrer [34] was used to determine bactericidal and bacteriostatic function of the peptides. Bactericidal activity of rhBD-2 (0.5 μg/5 μl) was determined by assays performed on gram negative *Escherichia coli* (BL21 DE3, *Invitrogen*) and *Pseudomonas aeruginosa* (ATCC strain 27853). Bacteriostatic activity was determined against gram positive *Staphylococcus aureus* (NCTC strain 8325). Bacterial isolates in appropriate media were grown overnight to mid-log phase, diluted to 4 x 10^6^ CFU/ml before further dilution and dispersion in a sodium phosphate-buffered trypticase soy broth-based low EEO agarose “underlay” (pH 7.4). A small diameter (2.5 mm) well for each sample was subsequently punched in the underlay gel, and a 5 μl aliquot of each sample applied to the gel. Samples contained 100 to 200 μg/ml rhBD-2 previously incubated in 100, 50 or 25 μM MGO, or phosphate-buffered saline (control). Plates were incubated aerobically at 37°C for 3 hours before returning the plates to room temperature and applying the trypticase soy broth-based agarose “overlay”. Plates were then incubated overnight at 37°C, and CFU within the zone of inhibition counted the following morning.

#### Chemotaxis assay

Chemotaxis assays were performed using the ChemoTx System from NeuroProbe with modification (NeuroProbe, Gaithersburg, MD). Briefly, samples of rhBD-2 were prepared at 3 concentrations (100 ng/μl, 200 ng/μl and 400 ng/μl) selected following kinetic analysis of optimum chemotaxis for CEM-SS cells, then incubated for 72 hr at 37°C in either 10μM MGO or 0.0067M PBS, pH 7.5. CEM-SS cells were initially grown in RPMI media, then resuspended in “chemotaxis media” (serum-free HG-DMEM with 1% BSA). The chemotactic mix containing rhBD-2 at final concentrations of 0, 10, 20 and 40 μg/ml, with or without MGO, or 10 nM stromal cell-derived factor 1 (SDF-1) as positive control (shown previously to induce a chemotactic response in CEM-SS cells, unpublished) were added to lower wells (30 μl) of a ChemoTx 96-well chamber. CEM-SS cell suspension was loaded into top wells of the chamber for a final cell concentration of 1 x 10^6^ cells/ml, and the chamber incubated for 2 hr in 5% CO_2_ at 37°C. After incubation CyQuant cell dye (Life Technologies, Grand Island, NY) was injected into the lower sample wells, and developing fluorescence quantitated using a BioTek microtiter plate reader (BioTek, Winooski, VT).

#### Statistics

Statistical analyses for antimicrobial and chemotactic functions were performed using exact probabilities for the nonparametric Mann Whitney ‘U’ Test. For antimicrobial function (bactericidal and bacteriostatic activity) N_1_ = 5, N_2_ = 5 (*E*. *coli*), or N_1_ = 6, N_2_ = 6 (*P*. *aeruginosa*, *S*. *aureus*). Chemotactic activity was determined in N_1_ = 3, N_2_ = 3 groups. Statistical significance is expressed as p = 0.05 or p = 0.01.

## Results

### MALDI-TOF MS mass detection of MGO- and GO-induced adduct formation on the rhBD-2 peptide

**Fig 1:**
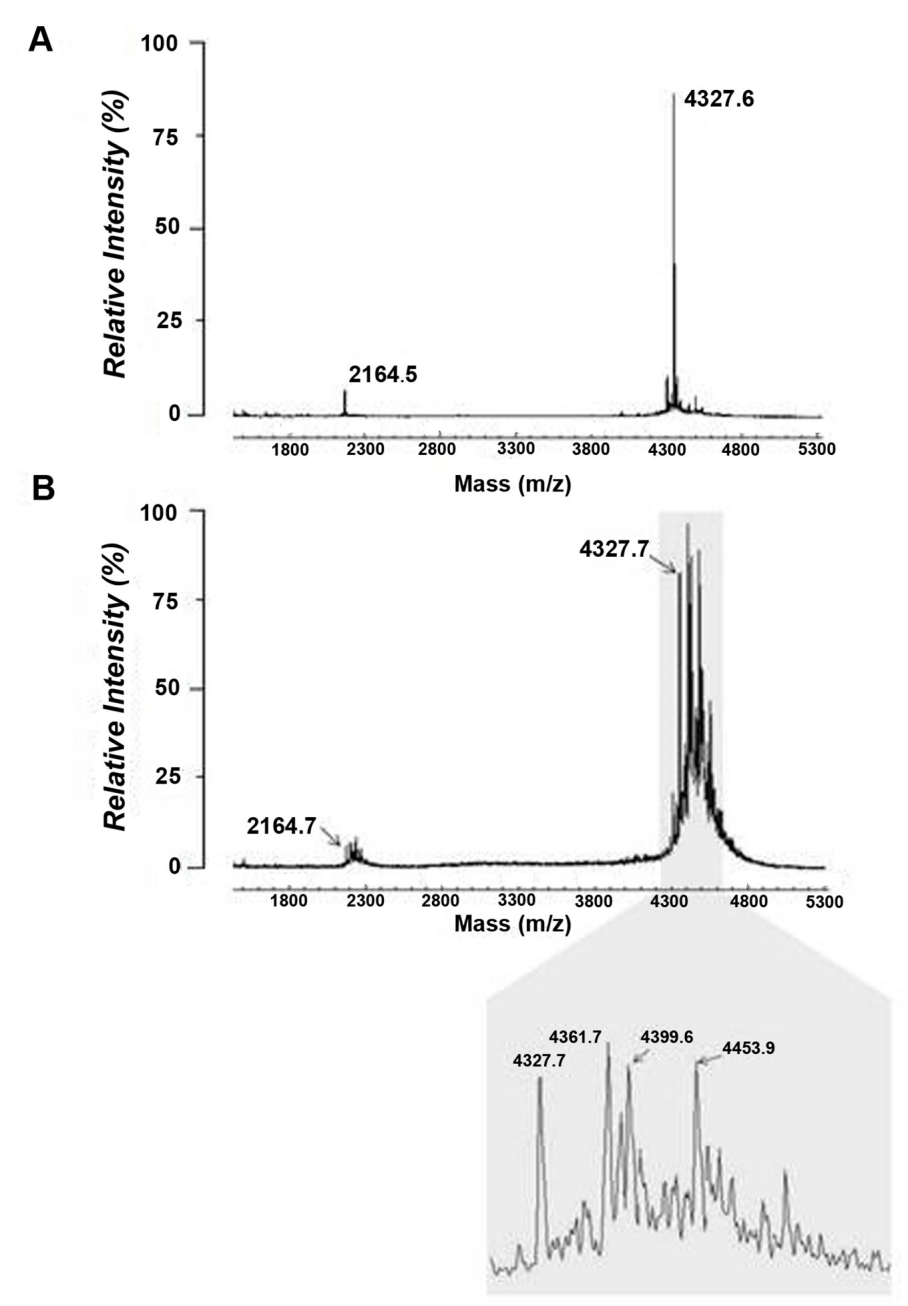
MALDI-TOF MS spectra obtained from native (untreated) rhBD-2 peptide (A) *vs* MGO-treated peptide (B). Native peptide exhibits singly and doubly protonated molecular ionic species at *m/z* 4326.6 and 2164.5, respectively. Exposure of rhBD-2 (20 ng/μl) to 100 μM MGO for 72 h resulted in additional peaks for both the singly and doubly protonated ionic species. Singly protonated ions (shaded area) show *m/z* increases of + 54 Da (*m/z* 4381.7), +72 Da (*m/z* 4399.7), and +126 Da (*m/z* 4453.7), suggesting adduction of dehydrated, intact, and dehydrated + intact MGO molecular species, respectively.

**Fig 2:**
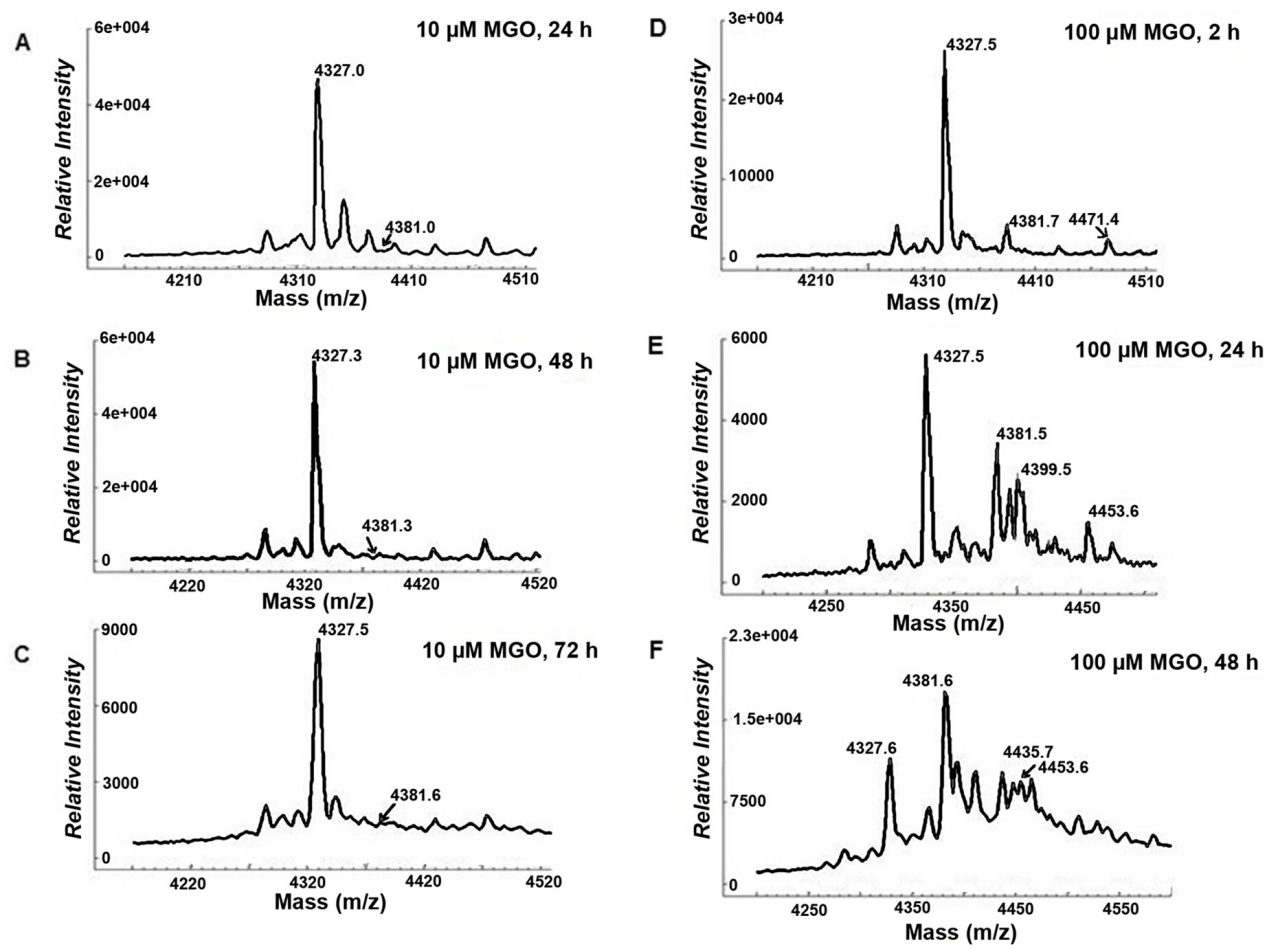
**A-C**. Expanded MALDI-TOF MS spectra obtained from rhBD-2 (20ng/μl) incubated at 37°C with 10 μM MGO for 24 h (A), 48 h (B), or 72 h (C) shows increasing peak intensity for a mass increase of + 54 Da (dehydrated) only with concomitant reduction of unadducted rhBD-2 peak intensity. **D-F**. Expanded MALDI-TOF MS spectra obtained from rhBD-2 (20ng/μl) incubated at 37°C with 100 μM MGO for 2 h (D), 24 h (E), or 48 h (F) shows increasing peak intensities with mass increases of + 54 Da (dehydrated), + 72 Da (intact), and combined ionic species of + 108 Da (+ 54 Da+ 54 Da), + 126 Da (+54 Da + 72 Da), and + 144 Da (+ 72 Da + 72 Da) with time of incubation. Increasing incubation time of the peptide with 100 μM MGO favors mass increases by dehydrated (+ 54 Da) ionic species, only with reduction of unadducted rhBD-2 peak intensity.

We used MALDI-TOF MS to determine if incubation of rhBD-2 with MGO or GO could induce mass changes in the peptide reflective of MGO- or GO-derived adducts. Untreated rhBD-2 incubated at 37°C in phosphate-buffered saline (PBS) for 72 h exhibited peaks at *m/z* of 4327.6 and 2164.5 (Fig 1A). These peaks corresponded to singly (1+) and doubly (2+) protonated rhBD-2 ionic species. Incubation of rhBD-2 in 100 μM MGO for 72 h, however, resulted in the appearance of additional peaks that corresponded in mass to molecular species of MGO (Fig 1B). These mass changes were equivalent to the mass of rhBD-2 protein adducted by intact MGO (*m/z* 4399.6), a dehydrated species (*m/z* 4381.7), or by a combined intact + dehydrated molecular species (*m/z* 4453.9) of MGO (Fig 1B, shaded area). Incubations less than 72 h and ranging from 2 h to 48 h also resulted in the appearance of additional peaks with increasing intensities at *m/z* of 4399.5, 4435.7 and 4453.6 (Fig 2a–2c). These masses corresponded to increases of +72 (intact), +108 (2 dehydrated), and +126 (1 dehydrated + 1 intact) representing MGO-derived adduction to rhBD-2 peptide. We observed a change in both peak profile and intensity when the molar concentration of MGO was reduced from 100 μM to 10 μM with incubation times of 24, 48 and 72 h (Fig 2d–2f). At 2 h incubation times, peaks corresponding to MGO-derived adducts were indistinguishable from background (data not shown). GO was far less reactive with the rhBD-2 peptide than MGO (S2 Fig). Nevertheless, mass increases of +40 (*m/z* 4367.7) and + 58 (*m/z* 4385.5), corresponding to dehydrated and intact GO-derived adducts were detected after incubation of the peptide to 100 μM GO for 48 and 72 h.

### MS/MS site identification of MGO modifications of hBD-2

**Fig 3:**
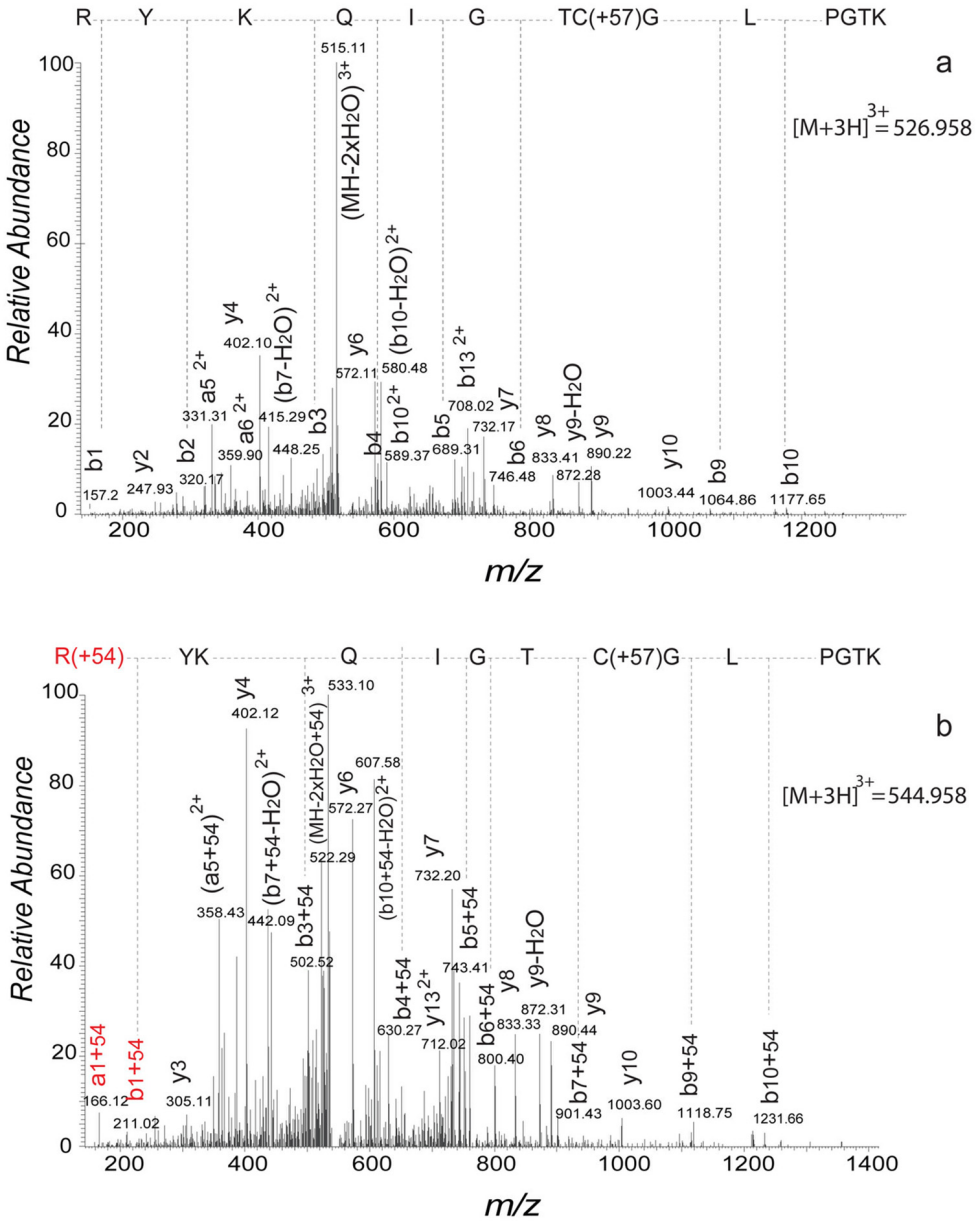
Comparison of deconvoluted tandom MS/MS spectra of untreated (a) vs modified (b) rhBD-2 peptide RYKQIGTCGLPGTK (23–36) after trypsin digestion of the rhBD-2 protein. The modified hBD-2 peptide was previously incubated in 100 μM MGO at 37°C for 72 h. The presence of the b1 ion with a +54 Da mass shift and unmodified doubly protonated y13 shows that modification of this peptide occurred at Arg^23^.

To determine the site of MGO modification, rhBD-2 was treated with dithiothreitol and then with iodoacetamide to reduce the three intramolecular disulfide bonds. Samples were then digested with trypsin and analyzed by LC-MS/MS as described in Methods. The ion signals at *m/z* 530.277 (2+), 467.556 (3+), 519.589 (3+), 526.951 (3+) and 566.294 (3+) that correspond to the unmodified peptides 1–10, 11–22, 11–23, 23–36 and 26–36, respectively, were observed by LC-MS analysis. The additional ion signals that correspond to a mass increase of +54 and +72 Da were detected only for peptides 11–23 and 23–36. Tandem MS analysis of these product ions revealed modification of the Arg^23^ residue by MGO (+72 Da) and by dehydrated MGO (+54 Da) adducts. Specifically, MS/MS analysis of triply protonated ion at *m/z* 544.955, corresponding to peptide 23–36 with a mass increase of +54 Da, produced a spectrum in which all the observed b-ions including a b_1_ ion were shifted by +54 Da (Fig 3b) relative to the corresponding control sample spectra of unmodified peptide 23–36 (Fig 3a). In contrast, all the observed y-ions, including doubly protonated y_13_ were unchanged. A similar fragmentation pattern was observed for triply protonated ion signal (at *m/z* 550.958) corresponding to peptide 23–36 with a mass shift of +72 Da. Furthermore, it was observed that all the b2-b7 and b10-ions were shifted by +72 Da (S3b Fig) while all the observed y-ions including doubly protonated y_13_ remained unmodified (S3a Fig). The MS/MS analysis of +54 and +72 Da adducts on peptide 11–23 of the rhBD2 protein showed a mass shift of +54 Da, corresponding to the modification of Arg^22^ (data not shown). An ion with a mass increase of +54 Da was the most prominent adduct observed for either the Arg^22^ or Arg^23^ peptide. Additionally, MGO adduction was observed on the N-terminus of the rhBD-2 protein in peptide 1–10. From the LC-MS/MS spectra of the doubly protonated ion (*m/z* 566.29), all observed b_2_-b_4_, b_6_ and b_9_ ions showed mass shifts of +72 Da, while observed y_4_ and y_6_-y_9_ ions remained unchanged. These observations indicate that modification of the peptide 1–10 by MGO (+72 Da) occurs at the N-terminal glycine (Gly^1^).

### Bactericidal and bacteriostatic activity of rhBD-2 is inhibited by MGO

**Fig 4:**
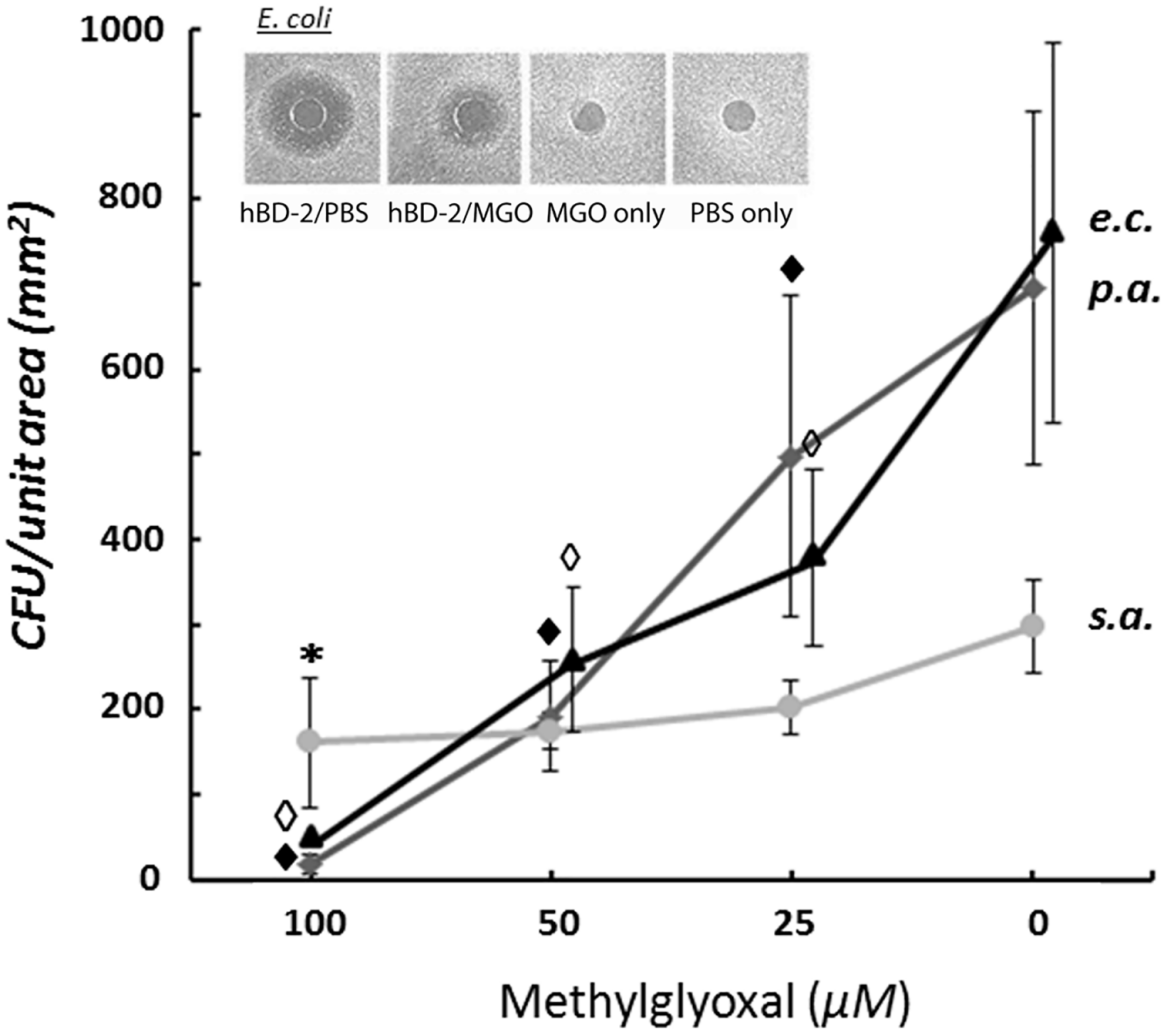
MGO-adducted rhBD-2 shows a concentration-dependent reduction of bactericidal activity, shown as a reduction in CFU vs unadducted peptide. CFU within a defined area were counted following radial diffusion assays performed with gram-negative, facultative anaerobes *Escherichia coli* (*e*.*c*.) and *Pseudomonas aeruginosa* (*p*.*a*.), and with the gram-positive, facultative anaerobe *Staphylococcus aureus* (*s*.*a*.) exposed to 0.5 μg/5 μl with or without MGO. Wild-type hBD-2 is highly bactericidal against most gram-negative bacterial strains, including *E*. *coli*, but this function is dramatically reduced following 72 h incubation of rhBD-2 (0.5 μg/5 μl) with 100 μM MGO at 37°C (inset). MGO (100 *μ*M) also reduces rhBD-2 bactericidal function against the gram-positive *S*. *aureus* strain. Data is presented as the Mean ± S.D. for N = 5 (*e*.*c*.) or N = 6 experiments (*p*.*a*., *s*.*a*.). Graph line for *e*.*c*. is offset slightly for clarity. (♦, ◊: p = 0.01; _*_: p = 0.05).

We show by radial diffusion assay [34] that bactericidal activity of rhBD-2 against gram-negative *P*. *aeruginosa* and *E*. *coli* is significantly reduced when either strain is grown in the presence of rhBD-2 exposed to the highest concentration of MGO (100 μM), and 25 μM MGO, the lowest shown (Fig 4). In several experiments we exposed the peptide to 5 uM MGO and still observed loss of bactericidal activity (data not shown). Bacteriostatic activity of rhBD-2 against gram-positive *S*. *aureus* was also adversely affected, but reduced activity reached statistical significance only when this strain was grown in the presence of rhBD-2 incubated with 100 μM MGO. Bacterial viability and growth rate were unaffected by the presence in the growth media of 100 μM MGO alone (Fig 4, inset).

### Chemotactic function of rhBD-2 is inhibited by MGO

**Fig 5:**
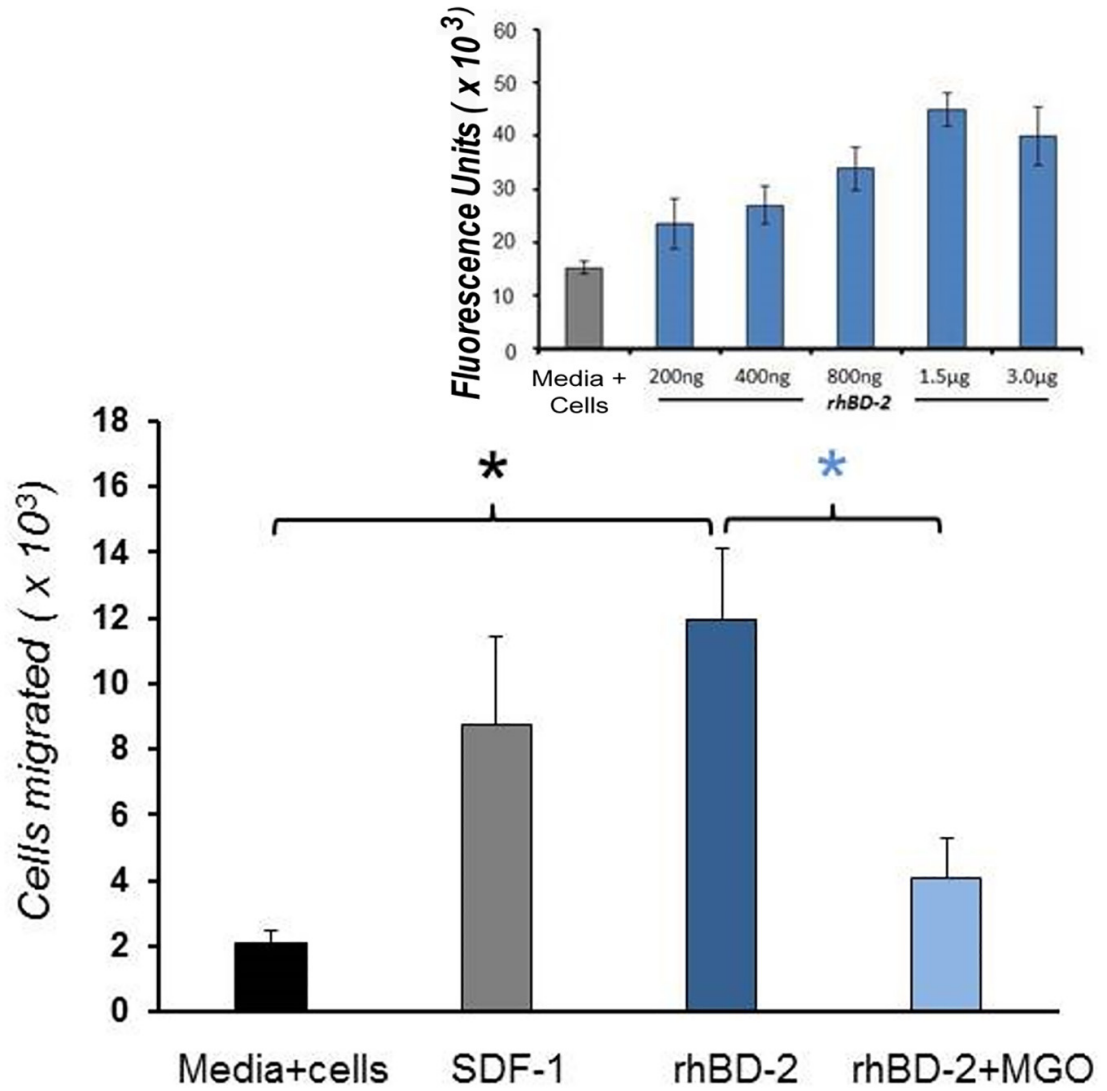
Effect of MGO adduction to rhBD-2 on chemoattraction for CEM cells. Inset, untreated rhBD-2 shows a concentration-dependent optimum in chemoattraction. Fluorescence units are proportional to migrated cell number due to labeling of migrating cell DNA by the CyQuant probe. Lower graph, chemoattractive function is reduced by incubating rhBD-2 with 100 uM MGO for 72 h at 37ଌ. SDF-1 is positive control for chemotaxis of CEM cells. Data is presented as the Mean ± S.E.M. for N_1_ = 3, N_2_ = 3 experiments _* *_: p = 0.05).

Because the hBD-2 peptide exhibits chemoattractive properties for CD4^+^ memory T cells [35, 36], we tested the ability of MGO-induced adducts of the rhBD-2 peptide to alter chemotactic function using the T-lymphoblastoid CEM-SS cell line. Both SDF-1 (positive control) and untreated (unadducted) rhBD-2 exhibited strong chemoattraction for our CEM test cells, with a direct correlation between rhBD-2 concentration and level of chemoattraction (Fig 5, inset). MGO-induced adduction of rhBD-2 resulted in a loss of chemotactic function that was observed beginning at rhBD-2 concentrations of 20 μg/ml and reaching statistical significance at 40 μg/ml (Fig 5). These results demonstrate that adduction of specific residues contributes to reduced chemotactic function by rhBD-2.

## Discussion

Beta-defensin-2 is an inducible member of the β-defensin family of antimicrobial/immunomodulatory peptides. These peptides are a prominent component of the human innate and adaptive immune systems not only through their ability to inhibit microbial invasion, but also through the capacity to modulate the adaptive immune response [37]. We show that antimicrobial function of rhBD-2 is severely compromised following *in vitro* exposure of the peptide to α-dicarbonyls at concentrations equivalent to tissue levels reportedly present under diabetic conditions [38]. Non-enzymatic glycation or Maillard reaction is known to be a significant contributor to the onset of hyperglycemia-induced pathologies associated with diabetes, and perhaps the chronic pathologies associated with aging and neurodegeneration [39]. Under physiological conditions production of dicarbonyl molecular species, notably methylglyoxal (MGO) and glyoxal (GO) occurs slowly, yet adduction to susceptible arginine (Arg) and lysine (Lys) residues can and does occur in both short-lived intracellular and long-lived extracellular proteins [40]. The impact of glycation on protein function depends, in part on the turnover rate of the protein, and the activity of the glyoxalase system responsible for degradation of unadducted dicarbonyl [41]. HBD peptides secreted by epithelial cells are generally resistant to degradation by proteases due to the presence of 3 intramolecular disulfide bonds, and appear to remain active for up to 4 days ([42], Ganz, personal communication). Chronic hyperglycemia associated with uncontrolled Type 2 diabetes, or an age-related disease will promote increased production of α-dicarbonyl molecular species, including highly reactive MGO. Normally, tissue levels of dicarbonyl remain low, limited primarily by activity of the glyoxalase system. In this way interaction between carbonyl and susceptible amino acid residues is largely prevented. However, depletion of glutathione by ageing, chronic hyperglycemia or associated oxidative stress reduces the ability of glyoxylase 1, a glutathione-dependent enzyme, to effectively limit dicarbonyl level [43, 44]. As a result, uncontrolled production of reversible Schiff’s base and Amadori intermediates that ultimately can convert to irreversible AGEs is a likely contributor to modification of the hBD-2 peptide with aging and in uncontrolled Type 2 diabetes. Under these conditions then α-dicarbonyl molecular species represent the dominant participants in protein modification. Modification of insulin with a plasma half-life of 5 to 10 minutes, considerably shorter than the apparent half-life of hBD-2, has been reported [45], although it has been proposed that modification of the protein most likely occurs within the pancreatic beta cell, and not subsequent to release into plasma. We have shown that *in vitro* exposure of rhBD-2 to MGO at a concentration as low as 10 μM, and for incubations as short as 2 h, results in detectible modification of Arg residues at positions 22 and 23. Despite the verified presence *in vivo* of MGO-modified proteins in serum, and hBD-2 secretion in human tissues, detection of the structurally modified peptide *in vivo* is difficult, most likely due to rapid removal of the adducted protein by mechanisms that recognize the modification as a signal for protein removal from the cellular environment. One such mechanism involves the irreversible modification of a single arginine residue by MGO, as we report here, and activation of a degradation process by monocytic cells that involves receptor recognition of the MGO-induced arginine derivative Ndelta-(5-hydro-5-methyl-4-imidazolon-2-yl)ornithine [46]. There is also evidence that the N-terminal residue may contribute to protein degradation in short-lived proteins [47]. However we did not determine whether the N-terminal glycine (Gly^1^) or its modification by MGO, as we observed, contributes to removal of rhBD-2.

Studies conducted both *in vitro*, and *in vivo*, show that in the presence of glycating agents like MGO, the mere presence of Arg and/or Lys residues within a protein is not predictive of non-enzymatic glycation at these sites. This point is well illustrated by Jia et al. [23], who report that *in vitro* insulin is susceptible to glycation by MGO, but glucagon is not, even though glucagon is of similar mass and contains two Arg and a single Lys. Additional support for the selective nature of non-enzymatic glycation of proteins comes from studies of human [48], mouse and rat [49] plasma proteins obtained from aged populations. In both these studies glycation is limited to relatively few proteins. Relative quantification of the extent of both the +54 and the +72 Da modification from LC-MS data shows that of the two Arg residues present in rhBD-2 Arg^23^ is slightly more reactive (21%) with MGO than Arg^22^ under our *in vitro* study conditions, this despite the close structural proximity of the two Arg residues. We note that in the 3-dimensional structure (S4A Fig) of the hBD-2 peptide Arg^22^, Arg^23^ and Gly^1^ are each found at or near the protein surface. A graphic comparison of residue solvent accessibility for hBD-2 (PDB 1FD3) shows both Arg^22^ and Arg^23^, as well as Gly^1^ located in the outer ring of the spiral plot, indicating these residues are readily accessible to MGO (S4B Fig). Protein tertiary structure, and therefore accessibility of the glycating agent to susceptible residues is a primary determinant of the glycation process. This is clearly shown by Gao and Wang [50] who correlated selective modification of specific arginine residues within the hemoglobin molecule with solvent accessibility, as determined by the ‘relative surface exposable area’ calculated for each arginine within the native molecule. In rhBD-2 both arginine Arg^22^ and Arg^23^, extending from the surface of the β1-β2 loop, are presumed to be equally solvent accessible. In native hBD-2 both residues are necessary to interactions between the peptide and prokaryotic membrane channel pores [51].

Our finding that adduction of both residues Arg^23^ and Arg^22^ contributes to reduced antimicrobial function of rhBD-2 against both gram negative (bactericidal activity) and gram positive bacteria (bacteriostatic activity) confirms the importance of these basic residues to antimicrobial function. The selective targeting of Arg residues by reactive carbonyl species, such as MGO [52] results in neutralization of the positively charged arginyl guanidino group [53] and the overall localized positive surface charge. Although we found that the N-terminal glycine (Gly^1^) also was modified by MGO, this region of the peptide does not appear to be a significant contributor to rhBD-2 antimicrobial function [54]. Several model membranes have been proposed to explain the antibacterial effects of hBD peptides, but common to each is the proposed insertion of a portion of the peptide into the bacterial lipid bilayer and dispersion of cellular integrity [55]. As shown by solid-state NMR distance measurements the positively charged Arg residue assumes a stabilizing configuration within the membrane through complexation of the positively charged Arg with the negatively charged phosphate groups of bacterial membrane lipids [56]. We therefore propose that modification of the guanidino group of the Arg residues, especially Arg^23^ by dicarbonyl contributes to reduction of positive surface charge, and destabilization of peptide-bacterial membrane lipid interaction.

Relevant to our findings are those of Pazgier and colleagues [57] demonstrating the critical nature of residues in the N-terminal α-helical region to CCR6-mediated chemotactic activity. In the Pazgier study mutations within the N terminus resulted in significant loss of hBD-1 affinity for the G protein coupled CCR6 receptor. Thus, although modification of the N-terminal glycine (Gly^1^) may not significantly influence antimicrobial function it might contribute to our observed reduction in rhBD-2 chemoattraction for CEM-SS cells. As pointed out by Pazgier et al. some residues located near the C terminus, such as Arg^29^ in hBD-1 also appear to influence chemotactic activity. Thus the Arg^22^ and/or Arg^23^ residues in rhBD-2 are likely participants in chemotactic activity as well, perhaps by complexing with glyosaminoglycans and dimerization of hBD-2 to initiate binding to the CCR6 receptor [58]. Adduction of these residues by dicarbonyls would effectively prohibit this interaction.

HBD-2 is one of several hBDs (1–4) important to the effective function of the human innate and adaptive immune systems. Utilizing mass spectral analysis of a recombinant antimicrobial peptide we have demonstrated that hBD-2 is susceptible to function-altering modification of critical Arg and Gly residues by dicarbonyl molecular species. Not reported here are additional findings from ongoing studies, indicating that modification of basic residues also occurs in other members of the hBD family, hBD-1 and hBD-3. In light of these findings we believe that in addition to hBDs, other cationic antimicrobial peptides with multiple Arg, such as cathelicidin (LL-37), may be equally prone to modification by carbonyls. The *in vitro* nature of our study findings are highly relevant to the *in vivo* conditions that exist in diabetes and other chronic conditions such as senescence and neurodegeneration where increased tissue levels of carbonyl contribute to protein modification. We describe here a previously unreported mechanism by which individuals with diabetes and other chronic diseases presenting with altered glucose metabolism may be at increased risk of chronic infection and tissue injury due to loss of effective antimicrobial and immunomodulatory protection. The clinical impact of MGO-modification on antimicrobial peptide function, and the inflammatory response *in vivo* will depend predominantly upon the frequency (probability) of dicarbonyl-antimicrobial peptide interaction. *In vivo*, these interactions will be influenced by multiple factors, including half-life of the peptide, concentration of the dicarbonyl in proximity to hBD-2, and competition for the dicarbonyl by other proteins. The clinical relevancy of our findings can only be determined by additional study *in vivo*.

## Supporting Information

S1 Fig Effect of 30 mM high glucose on hBD-2 mRNA expression in cultured human primary oral epithelial cells (HOEC) challenged with Fn cell wall (Fn). Results are shown as % of Control (challenge with 10 μg/ml Fn, normal glucose vs Fn challenge, high glucose). Scratch wounding had no detectable effect on hBD-2 expression (data not shown). However exposure of HOEC to high glucose for 24 h resulted in a marked reduction in hBD-2 mRNA expression, relative to the normoglycemic Control of ≥ 40%. Results are expressed as the mean ± S.D. of N = 5 independent experiments.
(TIF)

S2 Fig Expanded MALDI-TOF MS spectra obtained from rhBD-2 (20ng/μl) incubated at 37°C with 100 μM GO for 48 h (A), or 72 h (B), shows increasing peak intensity for mass increases of + 40 Da (dehydrated) and 58 (intact), along with a reduction in un-adducted rhBD-2 peak intensity. (TIF)

S3 Fig Comparison of deconvoluted MS/MS spectra of untreated (a) vs modified (b) rhBD-2 peptide RYKQIGTCGLPGTK (23–36) after trypsin digestion of the rhBD-2 protein. The modified rhBD-2 peptide was previously incubated in 100 μM MGO at 37°C for 72 h. The presence of the b2 ion with a +72 Da mass shift and unmodified doubly protonated y13 shows that modification of this peptide occurred at Arg^23^.
(TIF)

S4 Fig [A] 3D structure of hBD-2 dimer (PDB ID: 1FD3) showing the selected residues Gly^1^, Arg^22^ and Arg^23^ as grey sphere. [B] The spiral view shows amino acid residues of hBD-2 (PDB ID 1FD3), in the order of their solvent accessibility (determined using online server http://www.abren.net/cgi-bin/asaview/plot.cgi, Ahmed et al, 2004). Spiral plots are generated by sorting all residues by their relative solvent accessibility. The radius of the sphere representing each residue is proportional to the accessible surface area of that residue, thus enabling a visual estimate of more accessible residues. These residues are then arranged in form of a spiral, such that the inner residues in this spiral represent buried residues and more and more exposed residues come nearer to the outer ring of the spiral. Gly^1^, Arg^22^ and Arg^23^ (marked with open red circle) are all in the outer ring.
(TIF)
