# Supplementary figures and images for: Modification of β-Defensin-2 by Dicarbonyls Methylglyoxal and Glyoxal Inhibits Antibacterial and Chemotactic Function In Vitro
Source: PLoS One. 2015 Aug 5;10(8):e0130533. doi: 10.1371/journal.pone.0130533 (PMC4526640; doi:10.1371/journal.pone.0130533)

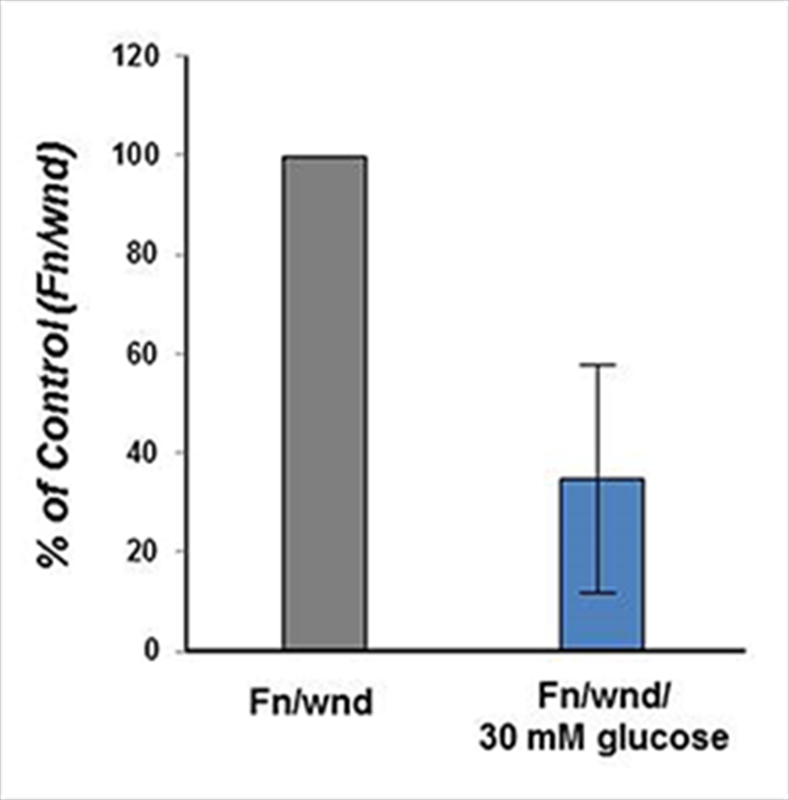

Supplement: S1 Fig — Results are shown as % of Control (challenge with 10 μg/ml Fn, normal glucose vs Fn challenge, high glucose). Scratch wounding had no detectable effect on hBD-2 expression (data not shown). However exposure of HOEC to high glucose for 24 h resulted in a marked reduction in hBD-2 mRNA expression, relative to the normoglycemic Control of ≥ 40%. Results are expressed as the mean ± S.D. of N = 5 independent experiments. (TIF) [file pone.0130533.s001.tif]

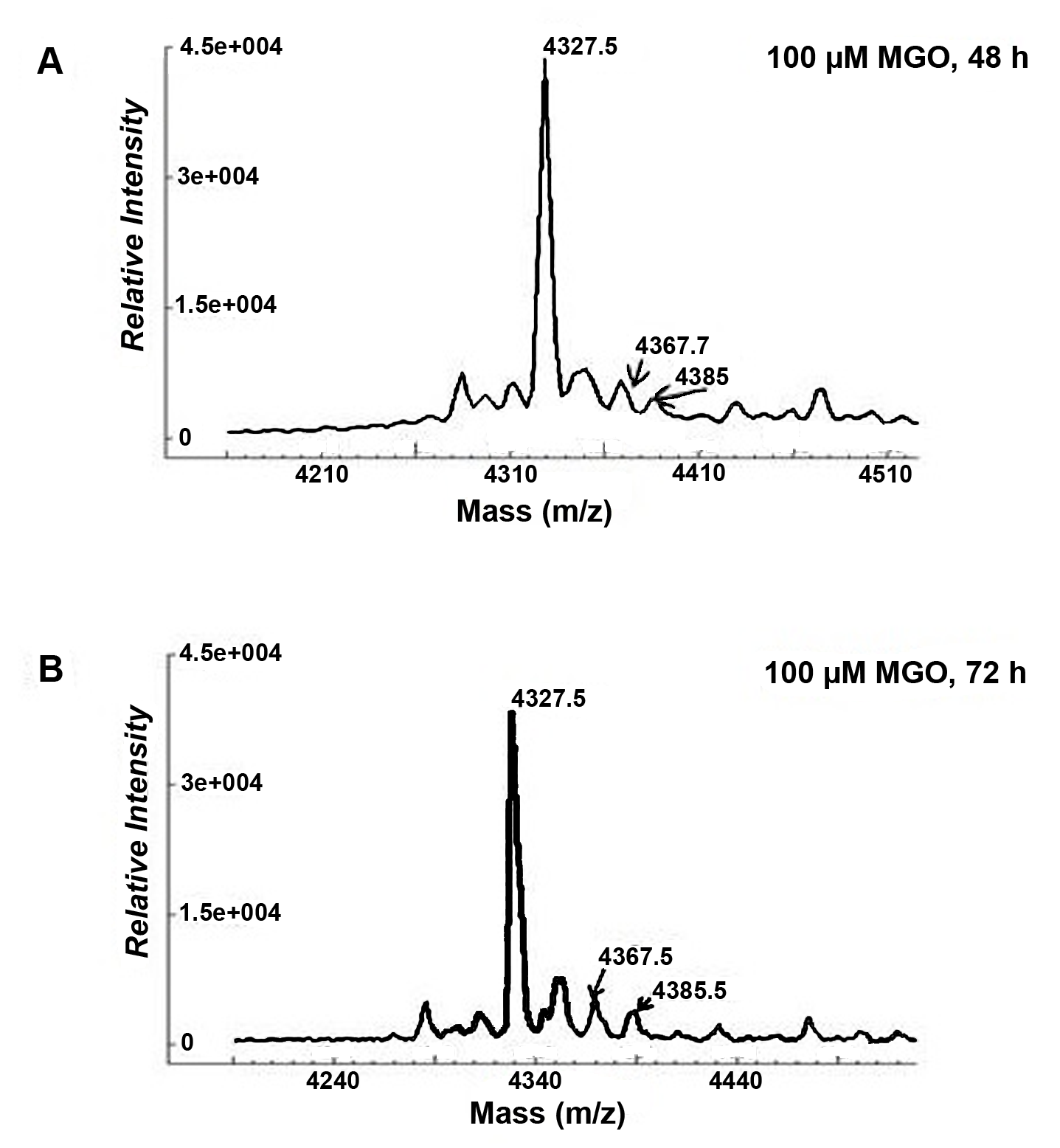

Supplement: S2 Fig — (TIF) [file pone.0130533.s002.tif]

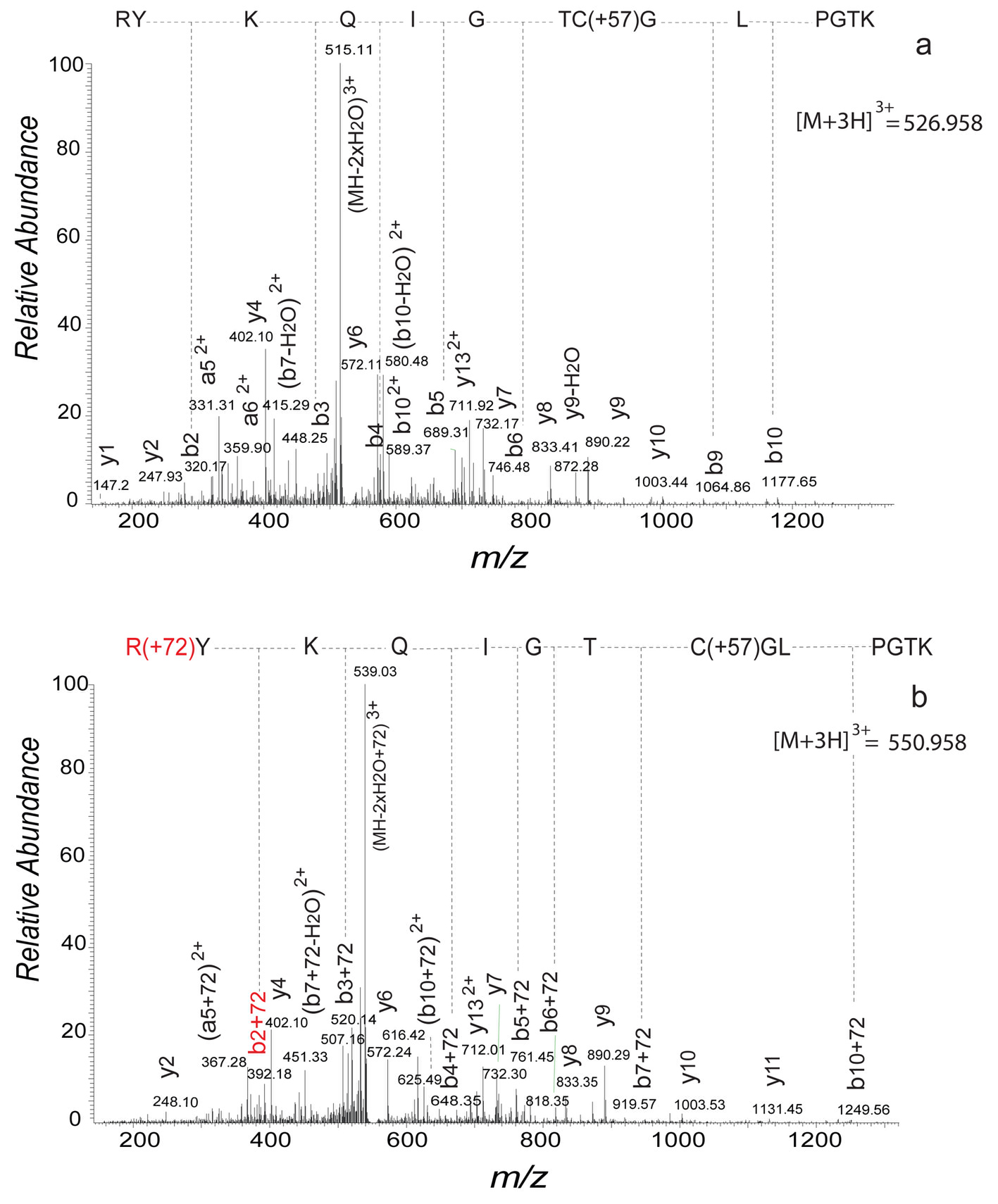

Supplement: S3 Fig — The modified rhBD-2 peptide was previously incubated in 100 μM MGO at 37°C for 72 h. The presence of the b2 ion with a +72 Da mass shift and unmodified doubly protonated y13 shows that modification of this peptide occurred at Arg23. (TIF) [file pone.0130533.s003.tif]
